# Recognising disease progression in MGUS and smouldering myeloma: Biomarkers, symptom monitoring and imaging

**DOI:** 10.1111/bjh.70492

**Published:** 2026-05-20

**Authors:** Aidan Haslam, Gulnaz Iqbal, Mark Drayson, Guy Pratt, Kwee Yong, Janet Dunn, Catherine Atkin, Chris Bunce, Donna Howe, Stella Bowcock

**Affiliations:** ^1^ School of Biosciences, College of Life and Environmental Sciences University of Birmingham Birmingham UK; ^2^ Department of Haematology University Hospitals Birmingham NHS Foundation Trust Birmingham UK; ^3^ Warwick Clinical Trials Unit, Warwick Medical School University of Warwick Coventry UK; ^4^ School of Infection, Inflammation and Immunology College of Medicine and Health Birmingham UK; ^5^ School of Medical Sciences, College of Medicine and Health University of Birmingham Birmingham UK; ^6^ University College London Cancer Institute University College London London UK; ^7^ Department of Haematology University College London Hospitals NHS Foundation Trust London UK; ^8^ Department of Acute Medicine University Hospitals Birmingham NHS Foundation Trust Birmingham UK; ^9^ Department of Haematology King's College Hospital NHS Foundation Trust London UK; ^10^ Faculty of Life Sciences and Medicine King's College London London UK

**Keywords:** biomarker, disease progression, imaging, monitoring, monoclonal gammopathy of undetermined significance (MGUS), myeloma, smouldering myeloma, symptom

## Abstract

The Tackling Early Morbidity and Mortality in Myeloma trial (TEAMM) trial recruited 977 newly diagnosed myeloma patients from 93 UK hospitals to assess the advantages and disadvantages of prophylactic antibiotics for the first 12 weeks. This paper analyses the 133 (14%) patients who had previously known precursor disease including monoclonal gammopathy of undetermined significance (MGUS) and smouldering myeloma (SMM), reporting the relative importance of blood‐based biomarkers, imaging findings and symptoms in their management. Prior to progression with active myeloma, patients with precursor conditions had symptoms for five times longer than patients with no diagnosed precursor phase. Although only 29% of patients reported new myeloma‐related symptoms developing after their first haematology appointment, 70% had a significant rise in paraprotein levels prior to progression. Likewise, risk scores for both MGUS and SMM increased prior to progression. Fractures occurred in 25% of patients despite being monitored for precursor disease. These fractures were difficult to predict as biomarkers lacked specificity and only 40% of patients reported back pain in combination with vertebral fractures. This study highlights the ongoing challenge of promptly recognising disease progression when using patient‐reported symptoms and monoclonal immunoglobulin monitoring.

## INTRODUCTION

Multiple myeloma (MM) is the second most common blood cancer^1^, ^2^, ^3^ with an estimated 188 000 new MM cases and 121 000 deaths worldwide in 2022.^4^ Patients with MM often experience long diagnostic delays^5^ and are frequently diagnosed in emergency settings with established organ damage.^6^, ^7^ MM has two main precursor conditions. Monoclonal gammopathy of undetermined significance (MGUS) is defined by the presence of a low‐level plasma cell clone as evidenced by a serum monoclonal antibody (<30 g/L) or abnormal serum free light chain studies combined with low‐level involvement of the marrow (<10% plasma cells). It has a constant rate of progression to active MM of slightly under 1% per year.^8^ Smouldering myeloma (SMM) is an intermediate stage with at least 10% plasma cells in the bone marrow or a paraprotein >30 g/L, but without MM defining criteria. It has a higher rate of progression to active MM of 10% per year for the first 5 years.^9^ The diagnosis of active MM differs from that of SMM by the presence of either organ damage or a pattern of biomarkers predicting imminent organ damage, collectively known as the SLiM criteria (*S*ixty percent or more clonal plasma cells within the bone marrow, involved *Li*ght chains >100 mg/L with a ratio of involved:uninvolved light chains >100 and >1 *M*RI defined focal lesio).^10^ Monitoring MGUS and SMM is important to reduce organ damage at progression and is thought to confer a survival advantage as a result.^11^, ^12^, ^13^ Monitoring includes regular measurement of paraprotein and free light chain levels, interval scanning and early symptom recognition.^14^ A deeper understanding of the relative importance and interrelationship between these variables is likely to better inform clinical practice and individual patient management. However, investigating the landscape of progression to active MM is challenging because the progression rate of MGUS is low and most studies focus on either patient‐reported symptoms, laboratory tests or imaging separately. To address these issues, we have studied a selected group of patients prior to receiving an MM diagnosis and entry into the Tackling Early Morbidity and Mortality in Myeloma trial (TEAMM).

TEAMM enrolled 977 individuals with newly diagnosed myeloma from 93 UK hospitals to receive anti‐myeloma treatment and 12 weeks of either levofloxacin or placebo.^15^ Of these, 149 (15%) participants were under the follow‐up of a haematologist for over 6 months prior to recruitment and so were thought to have precursor disease. We previously reported that these patients had fewer vertebral fractures (*p* = 0.001), fewer adverse features (*p* = 0.001), less decline in performance status (*p* = 0.001) and a lower stage (*p* = 0.04) at diagnosis than 813 participants with de novo MM.^11^ Here, we have examined antecedent symptoms reported by patients at progression which they thought were related to myeloma, and new data collected retrospectively on the blood and imaging tests performed during the precursor phase.

## METHODS

All analyses were performed using R statistical software version 4.4.3^16^; summary tables were made using the gtsummary R package^17^ with statistical analysis supported by the epitools R package.^18^ Unless otherwise stated, hypothesis testing of continuous data used the Mann–Whitney *U*‐test and categorical data used the Fisher's exact test. Confidence intervals (CIs) for risk ratios were calculated using the Wald method.

The process by which TEAMM recruitment data were collected is described in detail by Bowcock & Atkin et al.^11^ Ethical approval was provided by NHS Research Ethics Committee West Midlands and sponsorship by the Universities of Birmingham and Warwick. Informed consent was obtained from patients in line with the Declaration of Helsinki. TEAMM is registered by ISRCTN under the number ISRCTN51731976. Briefly, during the recruitment consultation, healthcare professionals asked ‘When did the patient first notice bodily changes and/or symptoms that they attribute to the myeloma?’ They were encouraged to write symptoms in the patient's own words, along with the date the symptoms occurred. Incidental findings of laboratory tests which led to hospital referral were also accepted. Bodily symptoms and incidental laboratory tests were analysed separately where appropriate. Demographic information and baseline clinical data were collected along with performance status 6 months prior to trial entry. The date of trial entry was taken as the date of diagnosis of active MM.

Patients under haematological follow‐up for 6 months or more prior to trial entry were presumed to have had a diagnosed precursor condition. Further information on initial diagnosis, biomarkers, imaging and management was requested from the 61 study sites for these patients. The case report form (CRF) is available in the Supporting Information. The start of precursor disease was defined as the earliest of the first bodily symptom, investigation related to myeloma or consultation leading to a diagnosis of precursor disease.

The revised definition of MM was published in 2014, which was midway through trial enrolment (August 2012 to April 2016).^10^ As a result, some patients under follow‐up for precursor disease would now be classed as having MM requiring treatment and these were excluded from analysis where possible. Data from the previously reported whole cohort and specifically the ‘de novo’ myeloma patients^11^ were compared with this cohort of patients with precursor disease.

## RESULTS

### Study population

**FIGURE 1:**
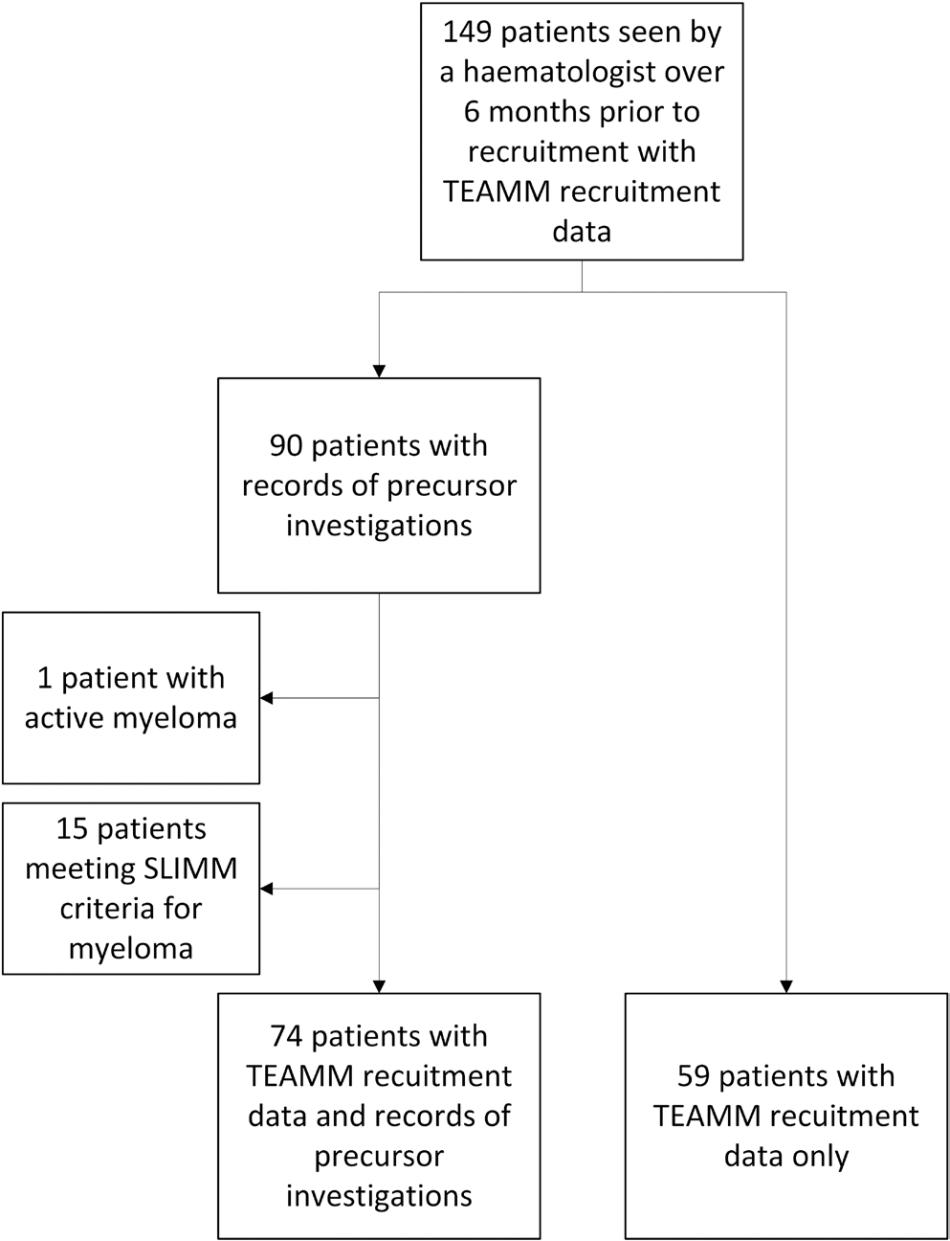
Consort diagram. Case report forms enquiring about the investigation and management of the precursor phase were sent to study sites for the 149 patients who had seen a haematologist at least 6 months before recruitment. Of those with completed forms, 15 were identified as having myeloma requiring treatment by SLiM criteria and one was excluded due to active symptomatic myeloma, leaving a total cohort of 133 patients.

One hundred and forty‐nine patients were under follow‐up for 6 months or more and 45 study centres returned CRFs for 90 of these individuals. The CRFs contained longitudinal results for haemoglobin (42 patients), paraprotein (51 patients) and light chain levels (30 patients) (Figure S3). Scan reports were returned for 76 patients. Fifteen patients who met SLiM criteria and one patient with active symptomatic MM were excluded leaving a total cohort of 133 TEAMM patients of whom 74 had records of precursor investigations and management (Figure 1). Of the 59 patients receiving haematology follow‐up but without completed CRFs, 71% (42) were recruited following the updated International Myeloma Working Group (IMWG) definition of MM compared to just 12% of patients with completed CRFs. Although SLiM MM cannot be ruled out in patients without CRFs, it is less likely given the timing of recruitment. There were no major differences in the performance status or demographics of patients with and without completed CRFs. Only 4% of patients were managed in primary care with the remainder followed up by haematologists. The median time from the diagnosis of precursor disease to trial entry was 730 days. The median symptom duration was used to estimate dates for two patients with missing data.

### Symptom reporting was influenced by patient awareness of precursor disease and gender

Sixty‐one per cent of patients with known or suspected precursor disease reported bodily symptoms that they attributed to MM, compared to 89% of patients with de novo MM (*p* < 0.00001).^11^ Table 1 shows key parameters in the 133 patients with precursor disease and is broken down according to the presence or absence of symptoms. Women were 20% more likely to report symptoms compared to men (*p* = 0.032). As 91% of the cohort was White British, the number of other ethnicities was too small to draw conclusions. Across both the known and suspected precursor disease groups, there was a consistent trend for the symptomatic group to suffer a deterioration in performance status prior to randomisation. Furthermore, among the patients reporting symptoms due to MM, the degree of marrow infiltration at diagnosis was greater. Whether symptoms attributed to myeloma were reported or not had no effect on precursor disease type at presentation, age or International Staging System (ISS) stage. As we have previously shown, patients with precursor disease have lower ISS scores at diagnosis than those with de novo MM^11^ and with further refinement of the precursor cohort, the differences are more stark (33% vs. 21% ISS 1, 52% vs. 35% ISS 2, 15% vs. 28% ISS 3 for precursor disease vs. de novo MM respectively).

**TABLE 1:** Cohort features based on the presence of absence of symptoms.

Characteristic	Overall *N* = 133^a^	No symptoms reported *N* = 52^a^	Symptoms reported *N* = 81^a^
Age	69 (61, 78)	72 (64, 79)	68 (61, 75)
Sex			
Female	60 (45%)	17 (33%)	43 (53%)
Male	73 (55%)	35 (67%)	38 (47%)
Precursor diagnosis at presentation to haematology^b^			
MGUS	41 (55%)	15 (56%)	26 (55%)
Plasmacytoma	2 (2.7%)	0 (0%)	2 (4.3%)
SMM	31 (42%)	12 (44%)	19 (40%)
Unknown	59	25	34
Ethnicity			
All other ethnicities	12 (9.0%)	1 (1.9%)	11 (14%)
White British/Irish	121 (91%)	51 (98%)	70 (86%)
ECOG at randomisation			
0	57 (45%)	27 (54%)	30 (39%)
1	55 (43%)	19 (38%)	36 (47%)
2	14 (11%)	4 (8.0%)	10 (13%)
3	1 (0.8%)	0 (0%)	1 (1.3%)
Unknown	6	2	4
ECOG decline during follow‐up	31 (25%)	8 (16%)	23 (30%)
Unknown	7	3	4
% Marrow plasmacytosis within 6 months of recruitment^b^	32 (20, 55)	26 (20, 40)	40 (25, 58)
Unknown	63	26	37
ISS			
1	36 (33%)	14 (30%)	22 (35%)
2	57 (52%)	25 (53%)	32 (51%)
3	17 (15%)	8 (17%)	9 (14%)
Unknown	23	5	18
Presence of vertebral fractures	28 (27%)	9 (25%)	19 (28%)
Unknown	29	16	13
Comorbidity score	3.00 (1.00, 5.00)	4.00 (2.00, 6.00)	3.00 (1.00, 4.00)
Unknown	27	8	19
Precursor duration (months)	280 (268, 288)	280 (270, 289)	278 (267, 286)

Abbreviations: CRF, case report form; ECOG, Eastern Cooperative Oncology Group; ISS, International Staging System; MGUS, monoclonal gammopathy of undetermined significance; SMM, smouldering myeloma.

^a^ Median (Q1, Q3); *n* (%).

^b^ Information present only in the precursor disease CRF (74 returned, see Figure 1).

Patients with a diagnosed or suspected precursor disease reported MM‐attributed symptoms for five times longer than those with de novo myeloma: 1.6 years (interquartile range [IQR] 1.1–5.0) vs. 0.3 years (IQR 0.1–0.6) respectively (*p* = 2.2 × 10^−16^). Eight per cent of our cohort reported symptoms from MM more than 5 years prior to diagnosis. Patients with SMM reported myeloma‐related symptoms for longer than those with MGUS (median 2.1 vs. 1.2 years).

**FIGURE 2:**
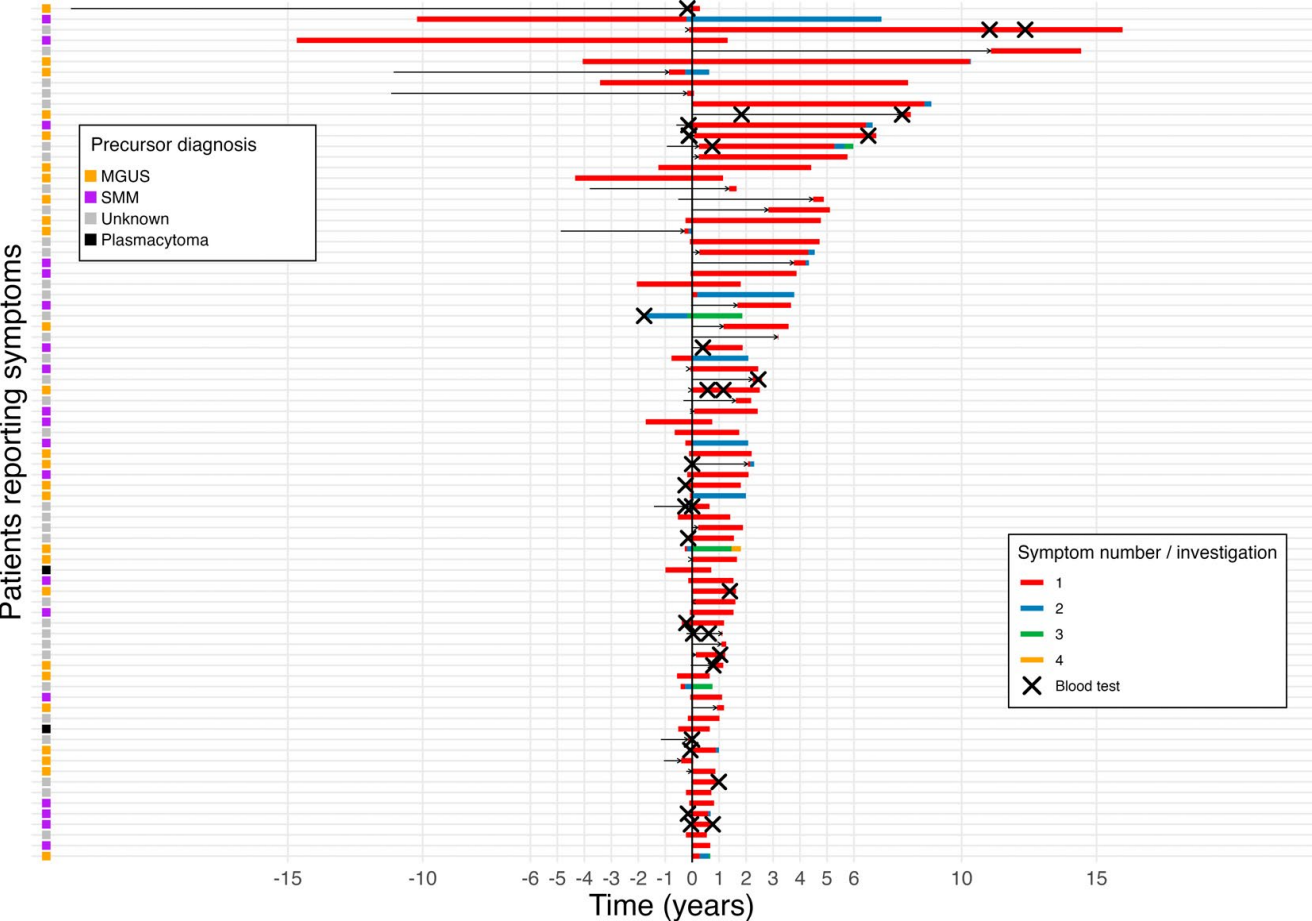
A swimmer plot of patients reporting bodily symptoms. Blood tests which led to a referral or intervention are shown as Xs. Time 0 is the first haematology appointment. Arrows show time passing from the start of precursor disease to the first symptom. Patient lines are coloured according to the diagnosis at the time of the first haematology appointment. Each patient line finishes at the date of MM diagnosis. MM, multiple myeloma.

During follow‐up with haematology, only 29% of patients reported new symptoms developing that they attributed to MM. There were no differences in the type of symptoms reported before compared with after the first haematology consultation (Figure S1). Progression to active MM did not occur at a constant rate during follow‐up as 56% of patients progressed to MM within 2 years of first seeing a haematologist (Figure 2, a version annotated with individual patient symptoms is available as Figure S2). Time to progression was independent of precursor disease type at first assessment, with both MGUS and SMM having a median precursor disease duration of 2.5 years (Figure 2).

### Biomarkers and risk scores

Most patients showed increases in paraprotein and light chain levels alongside a falling haemoglobin over time. However, paraprotein changes were not universal indicators of progression. Notably, 30% (19/63) of patients progressed with a peak paraprotein level less than 5 g/L above their value at presentation.

**FIGURE 3:**
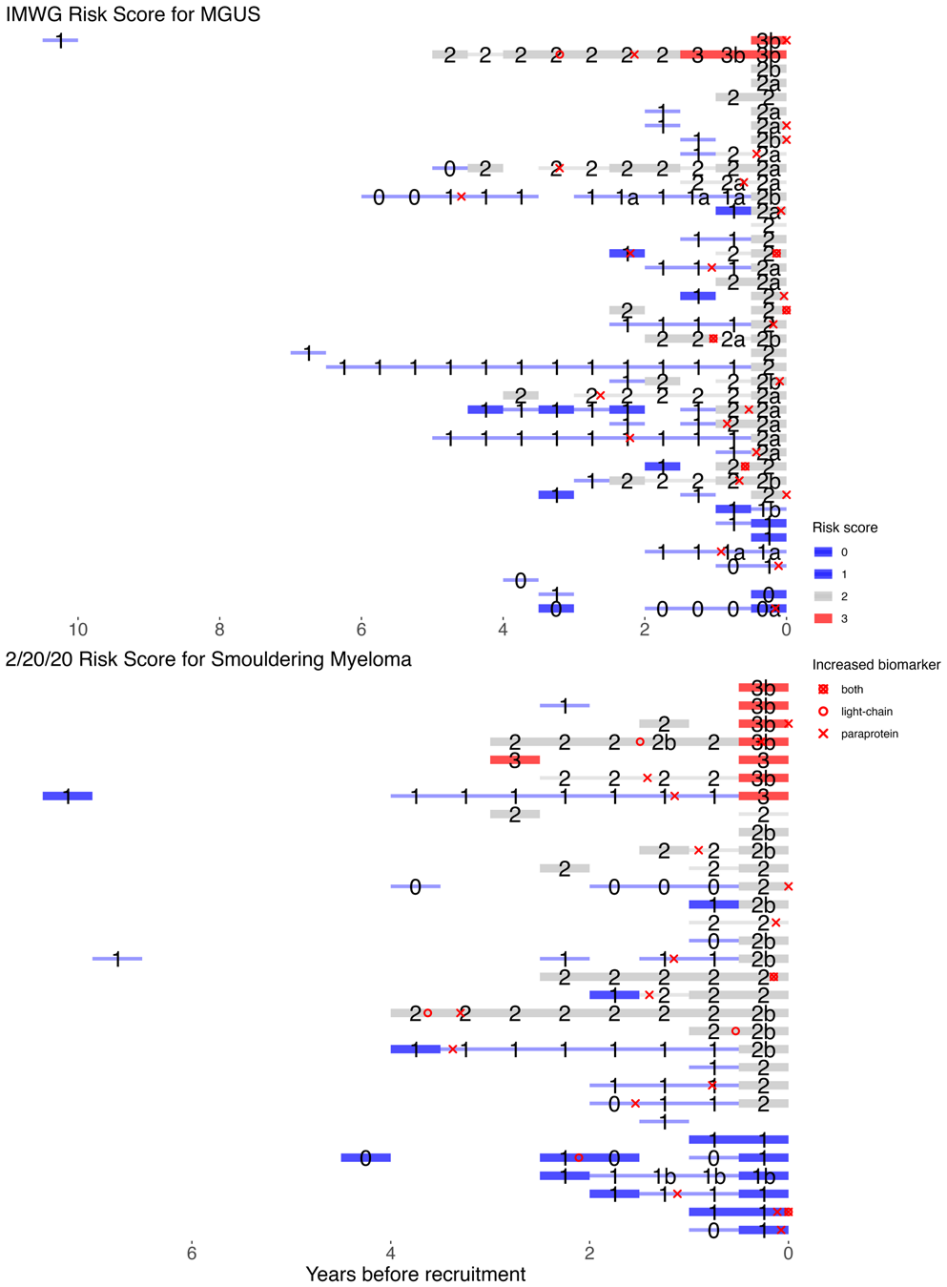
IMWG risk scores and their evolution over time for MGUS and smouldering myeloma. Thin and transparent lines are used to show time points in which serum free light chains were not measured. Increases in light chains and paraproteins of >100 mg/L and >5 g/L are shown using Os and Xs respectively. For MGUS patients developing results consistent with SMM (a paraprotein level > 30 g/L or bone marrow plasma cell infiltrate >10%), the suffix ‘a’ is applied to the risk score. For both MGUS and smouldering myeloma, the suffix ‘b’ indicates multiple myeloma defined by either bone marrow plasma cell percentage or light chain levels. MGUS, monoclonal gammopathy of undetermined significance; SMM, smouldering myeloma.

Consistent with the findings of Landgren et al.,^19^ the IMWG and 2/20/20 risk scores for MGUS and SMM, respectively, increased prior to progression to active MM. This was more likely with a greater duration of follow‐up (Figure 3). At the time of the study, serum free light chains were not widely used, and this prevented a longitudinal assessment of changes in risk score for 16 patients with MGUS and nine patients with SMM. Of the remaining MGUS patients, only 26% (5/19) had stable biomarkers and no interim bone marrow examination, producing no increase in risk score nor change in diagnosis to SMM. Forty‐two per cent of the MGUS patients (8/19) had an increase in risk score during follow‐up. Of the 13 SMM patients with sufficient investigations to enable a longitudinal assessment of SMM risk scores, five had an increase in score prior to progression with active MM. Interim bone marrows were performed in 8 of the 31 patients with SMM.

### Fractures occurred in a quarter of patients and were unpredictable

Combined data from TEAMM recruitment, CRFs and available scan reports identified fractures in 26% of patients (34/133). This likely underestimates the true prevalence, as scan reports were not available for the full cohort. Most fractures (*n* = 28) involved the spine. One patient had completed cauda equina syndrome and reported faecal incontinence, and another had retropulsion of bone fragments into the canal. Nine patients had pathological fractures at other sites including the femoral neck, lesser trochanter and radius.

Patient‐reported symptoms were less sensitive in precursor disease as only 40% of patients with spinal fractures reported back pain at any point, compared to 68% with de novo MM. Back pain was equally predictive, however, for spinal fracture in both patients with precursor disease and de novo MM (relative risk scores of 2.2, 95% CI [1.2–4.1] and 2.2, 95% CI [1.7–2.7] respectively). ISS score, precursor disease diagnosis (MGUS vs. SMM) and bone marrow plasmacytosis were uninformative and there were no obvious differences in trends of paraprotein, haemoglobin or involved light chains between those developing fractures and not (Figure S3). Patients with fractures had higher comorbidity scores (4, IQR 3–6) than those without (3, IQR 1–5), *p* = 0.032. This remained significant after removing the musculoskeletal component of the score, which would be influenced by a history of fractures.

**FIGURE 4:**
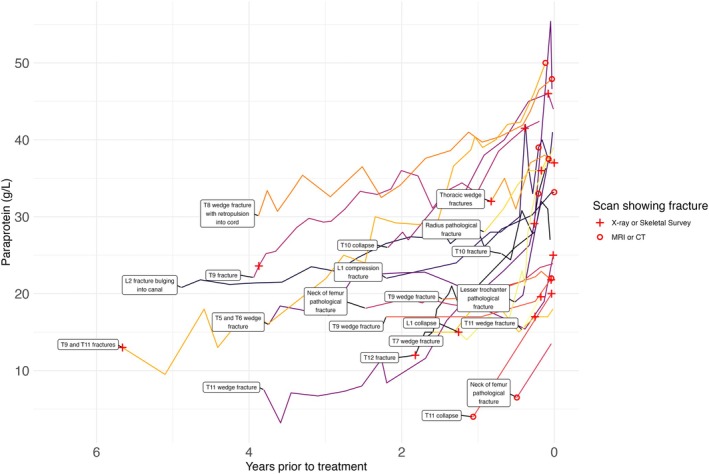
Paraprotein trends prior to treatment for the 21 patients with fractures and serial paraprotein results available. The pattern was heterogeneous, with some patients progressing for several years prior to fracture while others had a rapid increase or little change prior to fracture and progression.

Figure 4 shows the relationship between the paraprotein trend and the date of the scan demonstrating a fracture. There was heterogeneity with some patients clearly progressing prior to fracturing, while for others, the increase in paraprotein was either minimal or so rapid to make timely intervention challenging. The four spinal fractures detected at the beginning of follow‐up by skeletal survey were thought to be caused by osteoporosis or, in one case, by a plasmacytoma. Only the patient with plasmacytoma suffered a further non‐vertebral fracture at progression to active MM. There was no consistent pattern of change in symptoms and risk prior to fracture, and in 30%, neither changed before the event (Figure S4). The 15 patients with SLiM criteria MM had a much higher rate of fractures at 40% (6/15) and a shorter time to recruitment of 1.9 years, compared with the 133 patients with precursor disease (2.5 years).

## DISCUSSION

This study highlights the difficulty in promptly diagnosing progression to MM from precursor conditions. The tendency for patients to progress quickly after diagnosis with a precursor condition (Figure 2) raises the question of how accurately initial staging of paraproteinaemia took place and serves as an important reminder to avoid premature diagnostic closure during early follow‐up of patients with MGUS and SMM.

Our study is limited by the fact that symptoms were collected retrospectively from patients with newly diagnosed MM and therefore reliant on recollection at a time of stress. However, our findings suggest that long‐term symptom monitoring in precursor disease is unlikely to have a high yield for two reasons. First, in comparison to de novo MM, patients with precursor disease were 28% less likely to report MM‐related symptoms, and second, when reported, these are often already present around the time of the first haematology assessment; only 29% of patients reported new symptoms during follow‐up. However, the fact that patients with precursor disease reported symptoms five times longer than those with de novo MM suggests that patient education and self‐monitoring may have helped patients identify symptoms early.

In some cases, patients misattributed symptoms to MM. In particular, for the patients reporting symptoms related to myeloma many years prior to progression (up to 17 years prior), it is highly unlikely that these symptoms were related to active MM. O'Donnell et al. reported that patients with stable MGUS and SMM have a 31% prevalence of moderate to severe bodily symptoms, and like Maatouk et al., they reported similar levels of psychological distress between patients with stable precursor conditions and active MM.^20^, ^21^ More recently however, the iStopMM study group reported no increase in psychological distress as a result of MGUS diagnosis following screening,^22^ a finding that has now been supported in SECURE, a UK‐based MGUS study.^23^ O'Donnell et al. also reported that women were more likely to experience higher levels of anxiety than men and we found associated gender‐based differences in the likelihood of reporting bodily symptoms (Table 1).

For our analysis of biomarker trends and imaging results, we had to rely on incomplete data with 56% of our cohort having a completed CRF. However, the fact that 25% of patients with precursor diseases had fractures during progression to active MM shows that there is scope for improvement, particularly given that two patients sustained spinal cord injuries. The prevalence of fractures among patients with MGUS who progressed within the SEER cohort (recruited 1994–2005) was comparable, at 26%–30%.^24^ The failure to diagnose subtle early MM may partly be explained by the routine use of skeletal surveys, which are inferior in detecting early MM skeletal changes compared to whole body (WB) low‐dose CT, with WB magnetic resonance imaging (MRI) and positron emission tomography CT offering further improvements.^8^, ^9^ Within modern cohorts followed up according to IMWG imaging guidelines (advanced baseline imaging for all, with yearly WB‐MRI for SMM), a much lower prevalence of fractures is observed. Kastritis et al. (recruiting from 2014 to 2023) reported that, from a cohort of patients with SMM, there was only one fracture among 48 progressors.^25^ Although advanced imaging at baseline was not obligatory, Abdallah et al. (recruiting from 2013 to 2022) likewise reported only nine patients with fractures from 110 progressing from SMM,^26^ collectively stressing the advances made both through advanced and sequential imaging in preventing skeletal disease.^27^ Several trials have now investigated early treatment of high‐risk SMM, with a recent meta‐analysis showing an increase in overall survival and a reduction in the risk of progression to active MM.^28^, ^29^ Our findings support the role of advanced imaging modalities in preventing fractures and as earlier treatment of high‐risk SMM is adopted, skeletal fractures during precursor disease will become less of an issue. Unfortunately, however, access to these modalities remains low within the United Kingdom and less than half of surveyed centres in 2021 offered WB‐MRI, with skeletal survey being the first‐line investigation for MM in 39%.^30^

## CONCLUSION

This study reveals the challenges of symptom monitoring in patients with precursor disease. We found that they lacked sensitivity to predict fractures or progression as only 29% of patients reported new symptoms due to MM during follow‐up. There was also a risk of patients mis‐attributing symptoms to MM, though these data are limited in being retrospective. Biomarker changes were more informative and common; 7 of 10 patients had an increase of at least 5 g/L in paraprotein from baseline prior to progression. Despite follow‐up by haematologists, fractures occurred in a quarter of all patients with precursor disease prior to diagnosis of active MM and these were challenging to predict. Wider adoption of IMWG imaging guidelines for precursor conditions is a key intervention to reduce morbidity, though this requires greater access in the United Kingdom to advanced imaging modalities. Regular monitoring of blood tests alongside functional imaging (WB‐MRI or PET‐CT) should be at the core of directing investigations in those suspected of progression, with patient‐reported symptoms playing a supporting role.

## AUTHOR CONTRIBUTIONS

**Mark Drayson:** Conceptualization; supervision; writing – review and editing; project administration; funding acquisition. **Stella Bowcock:** Conceptualization; supervision; methodology; writing – review and editing; project administration. **Kwee Yong:** Writing – review and editing. **Donna Howe:** Project administration; data curation. **Gulnaz Iqbal:** Writing – review and editing; data curation; formal analysis; project administration; methodology; visualization. **Chris Bunce:** Supervision; writing – review and editing; visualization. **Aidan Haslam:** Methodology; software; formal analysis; visualization; writing – review and editing; writing – original draft; investigation; resources; data curation. **Janet Dunn:** Methodology; funding acquisition; data curation; project administration; writing – review and editing. **Catherine Atkin:** Project administration; data curation. **Guy Pratt:** Writing – review and editing; supervision.

## FUNDING INFORMATION

Funding was provided by the National Institute for Health and Care Research (14/24/04) and Blood Cancer (MRL02.23) UK. Additional funding was provided by Myeloma UK reference MUK2021.ED03.

## CONFLICT OF INTEREST STATEMENT

The authors have no conflict of interest to disclose.

## Supporting information


Data S1.

Figures S1–S4.

## Data Availability

Participant data are stored on a secure server at Warwick Clinical Trials Unit where each participant has been assigned a deidentified trial number. No identifiable data, such as name, address, hospital number, NHS number and date of birth, will be shared. Any requests for access to the data should be sent to wctudataaccess@warwick.ac.uk and agreement will be made through the data access committee. Requestors who are granted access to the data will be required to complete a data sharing agreement.
