# Supplementary material for: Recognising disease progression in MGUS and smouldering myeloma: Biomarkers, symptom monitoring and imaging
Source: Br J Haematol. 2026 May 20;208(6):2079–86. doi: 10.1111/bjh.70492 (PMC13267434; doi:10.1111/bjh.70492)
Supplement: Supplementary file 1 — Data S1. [file BJH-208-2079-s001.pdf]

MGUS/SMM Form

Participant Trial Number: ☐☐☐ Participant Initials: ☐☐ Date of Birth: d d - m o n - y y y y

Randomising Site: ☐

We are studying the impact of having a preceding diagnosis of MGUS or smouldering myeloma on presentation and outcomes. We need to clarify if your patient fits into these categories.

To be completed for any patients seen by a haematologist more than 6 months prior to entering the TEAMM trial who may have had a diagnosis of MGUS or smouldering myeloma

16.1 DIAGNOSIS DETAILS

1. When was the patient first seen by a haematologist? d d - m o n - y y y y

2. Was a diagnosis made as a result of this first visit:

No ☐ → Reason why diagnosis was not made

Yes ☐ → *If yes, please provide date of diagnosis and diagnosis type:* → d d - m o n - y y y y

3. Diagnosis type:

☐ Initial referral and diagnosis completely unrelated to suspected plasma cell disorder ☐ MGUS ☐ SMM ☐ Active myeloma (*please provide reason for deferring treatment)*

Reason for deferring treatment

16.2 LAB RESULTS AT THE TIME OF 1ST REFERRAL TO HAEMATOLOGY:

Date of test: d d - m o n - y y y y

1. Paraprotein type: IgG ☐ IgA ☐ IgM ☐ IgD ☐ Light chain only ☐

2. Immunology:

	Unmeasurable	Measurable	Value	Date if different from above OR tick box if same as above	
1. IgG (g/L)	☐	☐ →	☐☐ . ☐☐ →	d d - m o n - y y y y	☐
2. IgA (g/L)	☐	☐ →	☐☐ . ☐☐ →	d d - m o n - y y y y	☐
3. IgM (g/L)	☐	☐ →	☐☐ . ☐☐ →	d d - m o n - y y y y	☐
4. IgD (g/L)	☐	☐ →	☐☐ . ☐☐ →	d d - m o n - y y y y	☐
5. Sflc Kappa (mg/L)	☐	☐ →	☐☐☐☐☐ →	d d - m o n - y y y y	☐
6. Sflc Lambda (mg/L)	☐	☐ →	☐☐☐☐☐ →	d d - m o n - y y y y	☐
7. Sflc Kappa/Lambda ratio (mg/L)	☐	☐ →	☐☐☐ →	d d - m o n - y y y y	☐
8. Urinary protein concentration mg/L	☐	☐ →	☐☐☐☐ →	d d - m o n - y y y y	☐

3. Hb ☐☐ . ☐ g/dl OR ☐☐☐ . ☐☐ g/l → d d - m o n - y y y y

4. Was an ESR done? **No** ☐ **Yes** ☐ → ☐☐☐ mm/hr → d d - m o n - y y y y

5. Plasma viscosity done? **No** ☐ **Yes** ☐ → ☐ . ☐☐ mPas → d d - m o n - y y y y

6. Was a bone marrow done?

No ☐ **Yes** ☐ →

If yes, please *provide result:*

1. BM aspirate smear morphology: Plasma cells ☐☐☐ % ☐ N/K
2. BM aspirate flowcytometry : Plasma cells ☐☐☐ % ☐ N/K
3. BM trephine morphology: Plasma cells ☐☐☐ % ☐ N/K

→ Was immunostaining used? **No** ☐ **Yes** ☐

16.3 RADIOLOGY (IMAGING WHICH TRIGGERED THE 1ST VISIT TO HAEMATOLOGY OR AS RESULT OF THE 1ST VISIT:

1. Was a skeletal Survey, X-rays or any other imaging done? **No** ☐ **Yes** ☐ → *Please indicate type of imaging, date performed and* ***attach a copy of the report***

Type of imaging:
→ X-Ray ☐ MRI ☐ PET ☐ CT ☐ PET-CT ☐
→ Date: d d - m o n - y y y y

16.4 MONITORING:

1. Who followed up these patients? **Haematologist** ☐ **GP** ☐

2. Was there a rise in any of the following over time?

a. Paraprotein **No** ☐ **Yes** ☐ b. SFLC **No** ☐ **Yes** ☐ c. Urinary protein concentration **No** ☐ **Yes** ☐

3. Do you have serial paraprotein, FLC levels, Hb and ESR/Plasma viscosity (PV) over this time interval? **No** ☐ **Yes** ☐ → Please **attach copies of the blood reports**)

16.5 TREATMENT:

1. What triggered the haematologist to start treatment?

a. Rise in paraproteins OR light chains	**No** ☐	**Yes** ☐
b. Patient reported symptoms	**No** ☐	**Yes** ☐
c. The appearance of a myeloma defining event (e.g. presence of bone lesions, renal failure, anaemia, hypercalcaemia, marrow infiltration ≥60%, sflc ratio >100)	**No** ☐	**Yes** ☐
d. Did the patient have further work-up?	**No** ☐	**Yes** ☐ → Please provide details below:

16.6. FURTHER WORK-UP DETAILS:

1. Bone marrow **No** ☐ **Yes** ☐ → Date: d d - m o n - y y y y

a. BM aspirate smear morphology: Plasma cells	☐☐☐ %	☐ N/K
b. BM aspirate flowcytometry : Plasma cells	☐☐☐ %	☐ N/K
c. BM trephine morphology: Plasma cells	☐☐☐ %	☐ N/K → Was immunostaining used? **No** ☐ **Yes** ☐

2. Bloods in the month prior to randomisation **No** ☐ **Yes** ☐

a. Hb			☐☐ . ☐ g/dl OR ☐☐☐ . ☐☐ g/l	→ d d - m o n - y y y y
b. Was an ESR done?	No ☐	Yes ☐ →	☐☐☐ mm/hr	→ d d - m o n - y y y y
c. Plasma viscosity done?	No ☐	Yes ☐ →	☐ . ☐☐ mPa	→ d d - m o n - y y y y
d. Creatinine	No ☐	Yes ☐ →	☐ . ☐☐ μmol/l	→ d d - m o n - y y y y
e. Calcium	No ☐	Yes ☐ →	☐ . ☐☐ mmol/l	→ d d - m o n - y y y y

3. Skeletal survey, X-rays or any other imaging done in the six months prior to randomisation? **No** ☐ **Yes** ☐ → *Please indicate type of imaging, date performed and* ***attach a copy of the report***

Type of imaging:
→ X-Ray ☐ MRI ☐ PET ☐ CT ☐ PET-CT ☐
→ Date: d d - m o n - y y y y

4. Other investigation *(please provide details)*: → Date: d d - m o n - y y y y

Other investigation details

Please **attach copies of the reports**

Form completed by (print name): ______________________ *(Please note: your name must be on the trial delegation log)*

Signature: ______________________ **Date** d d - m o n - y y y y
