# Supplementary material for: Recognising disease progression in MGUS and smouldering myeloma: Biomarkers, symptom monitoring and imaging
Source: Br J Haematol. 2026 May 20;208(6):2079–86. doi: 10.1111/bjh.70492 (PMC13267434; doi:10.1111/bjh.70492)

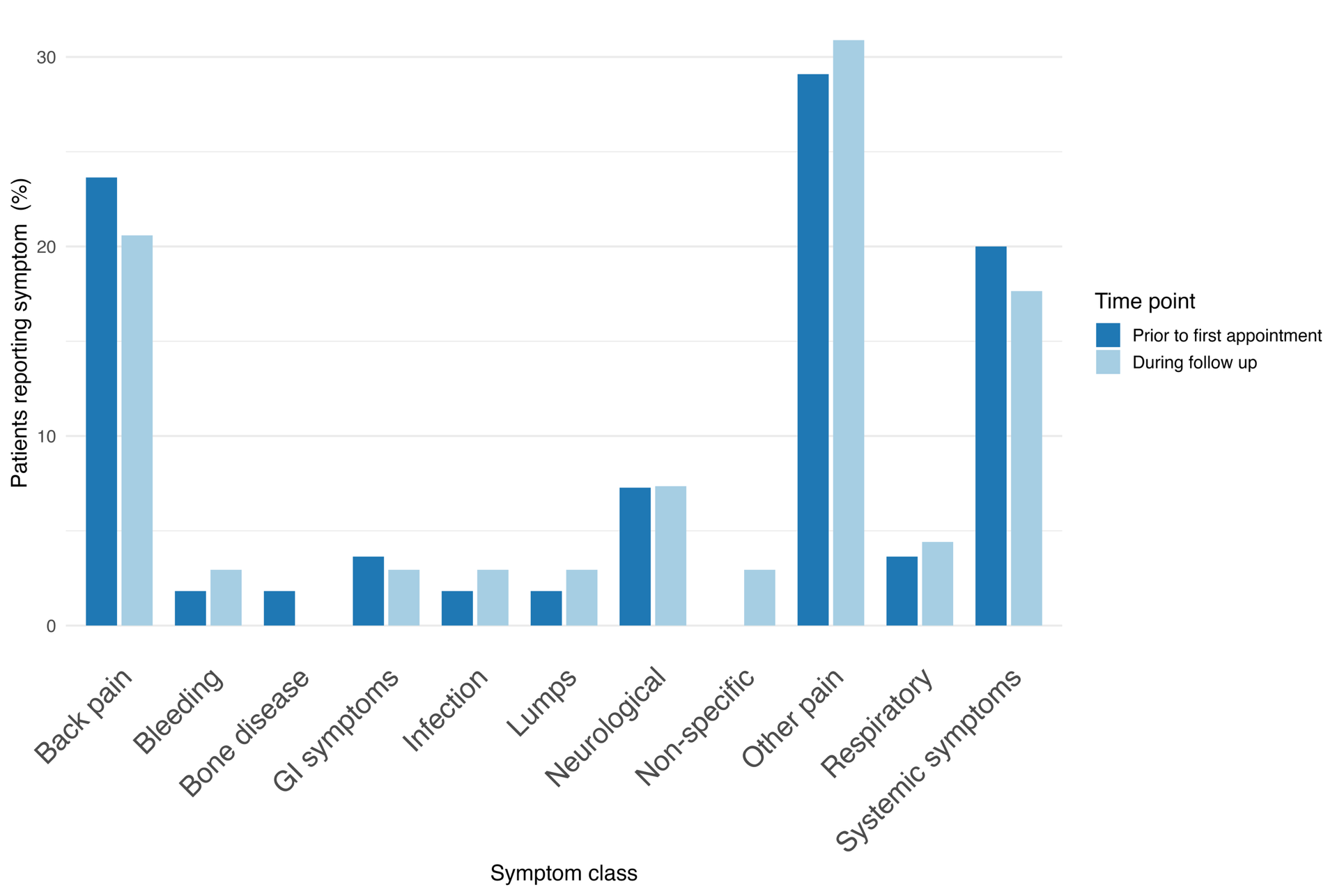


Supplementary Figure 1 Proportion of new symptoms reported prior to the first haematology appointment compared to during haematology follow up. 46 patients reported symptoms related to MM prior to their first haematology appointment and 38 patients reported new symptoms related to MM after their first appointment.

*Supplementary figure 2 A swimmer plot of the 82 patients reporting symptoms. The solid line represents the first haematology appointment. Patient-reported symptoms are annotated on the plot according to when they first started.*


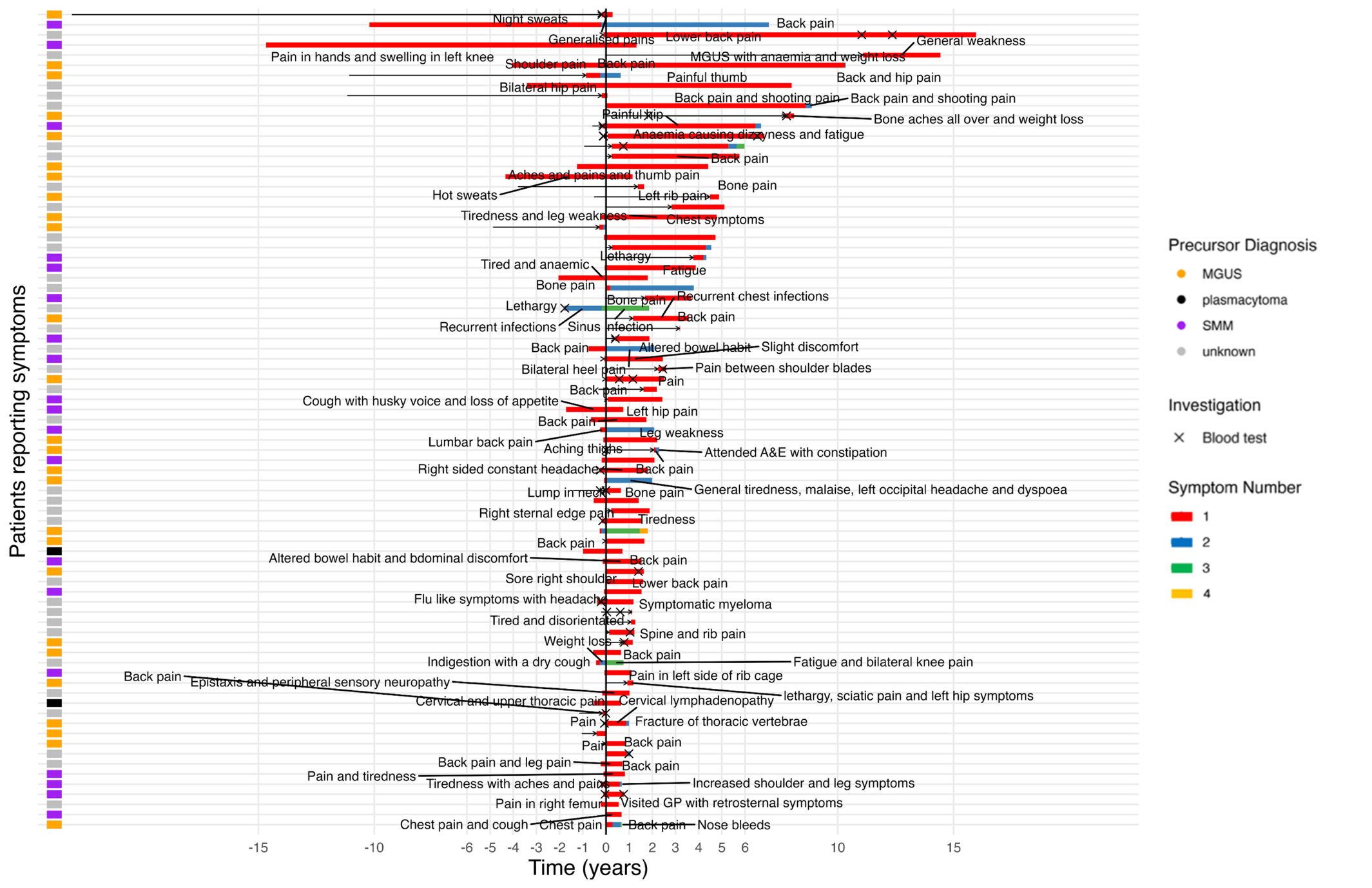


*Supplementary figure 3 Patients with fractures were separated into three groups based on whether a change in symptom, biomarker or neither preceded the occurrence of a fracture. The score is either the IMWG or 2/20/20 risk score for MGUS and SMM respectively. A cut-off of 5g/L and 100mg/L for changes in paraprotein and light chains were chosen to signify a significant increase.*


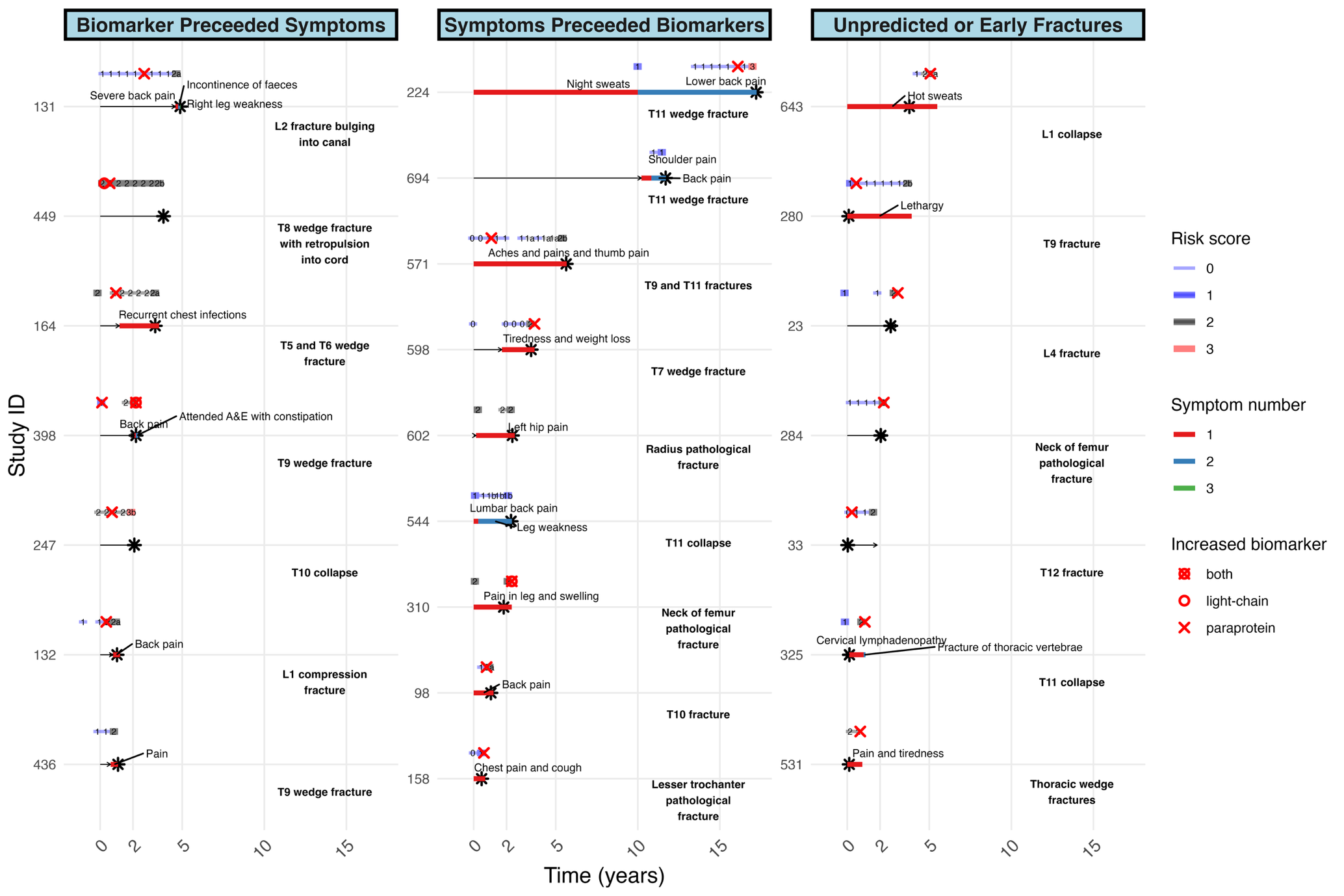


Supplementary Figure 4 Muted spaghetti plots of paraprotein, haemoglobin and involved serum free light chain trends prior to treatment with chemotherapy or recruitment, whichever occurred first. All data are presented on both sides but highlighted blue according to fracture status to facilitate comparison. When biomarkers improve prior to chemotherapy, this may be due to steroid treatment which was allowed in the TEAMM protocol.


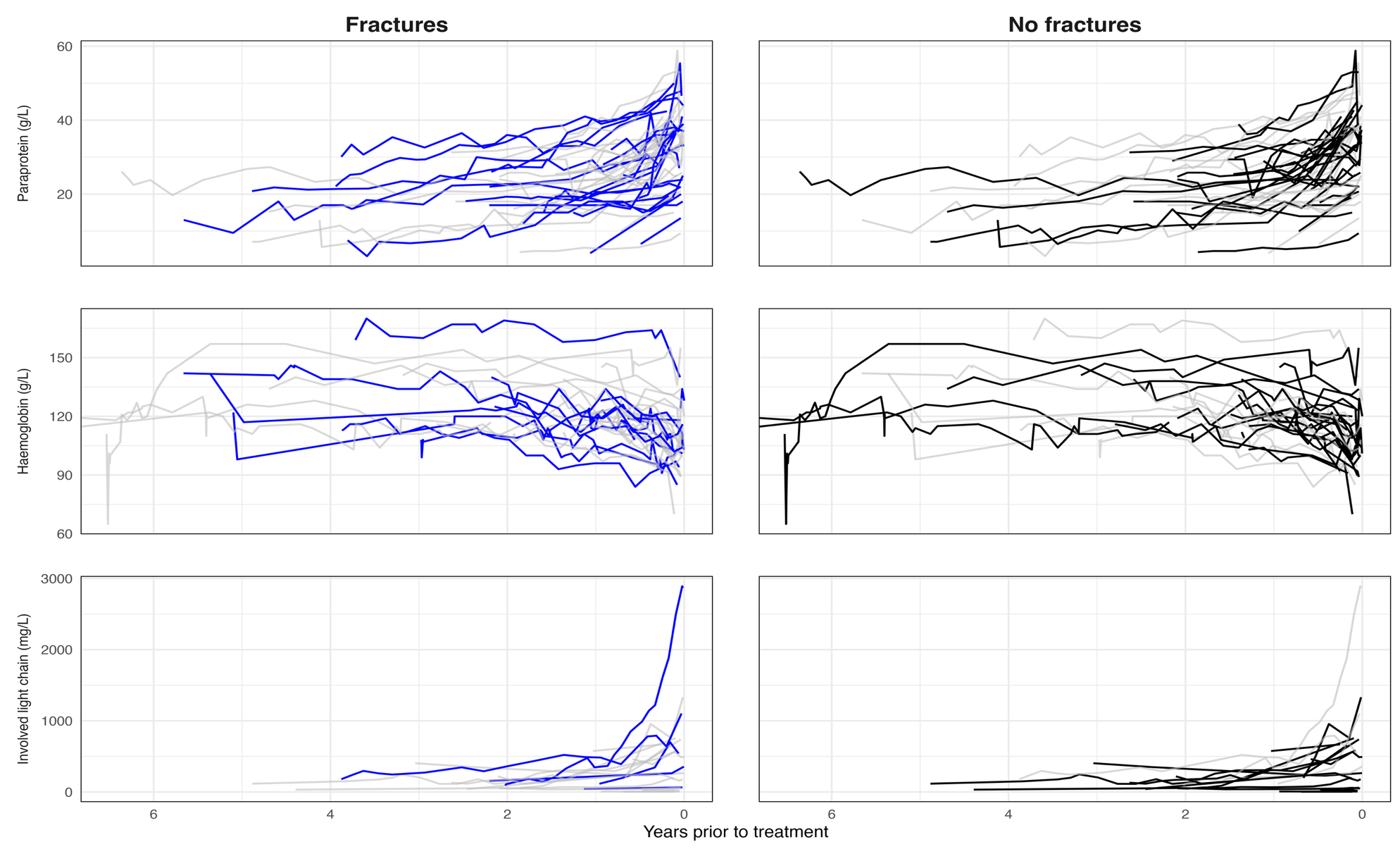

Supplement: Supplementary file 2 — Figures S1–S4. [file BJH-208-2079-s002.docx]
